# Typhoid intestinal perforations at a University teaching hospital in Northwestern Tanzania: A surgical experience of 104 cases in a resource-limited setting

**DOI:** 10.1186/1749-7922-7-4

**Published:** 2012-03-08

**Authors:** Phillipo L Chalya, Joseph B Mabula, Mheta Koy, Johannes B Kataraihya, Hyasinta Jaka, Stephen E Mshana, Mariam Mirambo, Mabula D Mchembe, Geofrey Giiti, Japhet M Gilyoma

**Affiliations:** 1 Department of Surgery, Catholic University of Health and Allied Sciences-Bugando, Mwanza, Tanzania; 2 Department of Internal Medicine, Catholic University of Health and Allied Sciences-Bugando, Mwanza, Tanzania; 3 Department of Microbiology and Immunology, Catholic University of Health and Allied Sciences-Bugando, Mwanza, Tanzania; 4 Department of Surgery, Muhimbili University of Health and Allied Sciences, Dar Es Salaam, Tanzania

**Keywords:** Typhoid fever, Intestinal perforation, Surgical management, Prognostic factors, Tanzania

## Abstract

**Background:**

Typhoid intestinal perforation is still prevalent in many developing countries. Despite the advances in the management, the outcome in these patients in resource limited countries is still very poor. This study was to review our experiences on the surgical management of typhoid intestinal perforation and to determine the prognostic factors for mortality in our local setting.

**Methods:**

This was a combined retrospective and prospective study of patients who were operated for typhoid intestinal perforation at Bugando Medical Centre between August 2006 and September 2011. Data collected were analyzed using SPSS computer software version 15.

**Results:**

A total of 104 patients were studied representing 8.7% of typhoid fever cases. Males were affected twice more than the females (2.6:1). Their ages ranged from 8 to 76 years with a median age of 18.5 years. The peak age incidence was in the 11-20 years age group. Fever and abdominal pain were the most common presenting symptoms and majority of the patients (80.8%) perforated between within 14 days of illness. Chest and abdominal radiographs revealed pneumoperitonium in 74.7% of cases. Ultrasound showed free peritoneal collection in 85.7% of cases. Nine (10.2%) patients were HIV positive with a median CD4+ count of 261 cells/μl. The perforation-surgery interval was more than 72 hours in 90(86.5%) patients. The majority of patients (84.6%) had single perforations and ileum was the most common part of the bowel affected occurring in 86.2% of cases. Simple closure of the perforations was the most commonly performed procedure accounting for 78.8% of cases. Postoperative complication rate was 39.4% and surgical site infection was the most frequent complication in 55.5% of cases. Mortality rate was 23.1% and it was statistically significantly associated with delayed presentation, inadequate antibiotic treatment prior to admission, shock on admission, HIV positivity, low CD4 count (< 200 cells/μl), high ASA classes (III-V), delayed operation, multiple perforations, severe peritoneal contamination and presence of postoperative complications (*P *< 0.001). The median overall length of hospital stay was 28 days.

**Conclusion:**

Typhoid intestinal perforation is still endemic in our setting and carries high morbidity and mortality. This study has attempted to determine the factors that statistically influence mortality in typhoid perforation in our environment. Appropriate measures focusing at these factors are vital in order to deliver optimal care for these patients in this region.

## Background

Typhoid fever, a severe febrile illness caused primarily by a gram negative bacillus *Salmonella typhi*, has continued to be a public health problem in many developing countries [1,2]. Typhoid infection is generally transmitted by faeco-oral route and may occasionally lead to an epidemic, particularly in areas with poor sanitation and limited availability of clean, potable water [1-4]. It is a global health problem that can have a devastating impact on resource-poor countries like Tanzania and it is estimated that more than 33 million cases of typhoid fever occur annually causing more than 500,000 deaths [2,5,6]. While control of the infection has been achieved in developed countries by effective public health measures, developing countries continue to bear the burden of the disease, principally because many communities still fall short of standards for drinking water, hygiene and sanitation [2,7,8].

The surgical complications of typhoid fever are a cause of significant morbidity and mortality in many parts of Africa, particularly in sub-Saharan Africa where standard medical facilities are not yet readily available

[4,6]. Intestinal perforation is a serious complication of typhoid fever and remains a significant surgical problem in developing countries, where it is associated with high mortality and morbidity, due to lack of clean drinking water, poor sanitation and lack of medical facilities in remote areas and delay in hospitalization [9]. The rates of perforation have been reported in literature to vary between 0.8% and 18% [10-13]. The high incidence of perforation in most developing countries has been attributed to late diagnosis and the emergence of multi-drug resistant and virulent strains of *Salmonella typhi *[14]. The disease affects mostly young adults who contribute enormously to the economy of third world countries [14-16]. It also affects children and it is most common in people in the low socio-economic strata [15].

The management of typhoid intestinal perforation poses diagnostic and therapeutic challenges to general surgeons practicing in resource-limited countries [6,15]. Surgery is considered the treatment of choice in order to improve the chances of survival of patients with this condition, who most often present late [17]. The management of these patients provides a number of unique challenges to the attending surgeon. Many of these patients present at and are managed in rural hospitals where resources are often very limited. The outcome of treatment of typhoid intestinal perforation may be poor especially in developing countries where late presentation of the disease coupled with lack of clean drinking water, poor sanitation, lack of diagnostic facilities and emergence of Multi-drug resistant (MDR) strains of *S. typhi *resulting from inappropriate and indiscriminate use of antibiotics are among the hallmarks of the disease [6,18]. Late presentation, inadequate preoperative resuscitation, delayed operation, number of perforations and the extent of fecal peritonitis have been found to have a significant effect on prognosis [19,20].

While mortality in the developed world has dropped to between 0% and 2% [21,22], mortality in the developing world remains high at between 9% and 22% [14,15,23]. The reasons for this state of affairs have not been evaluated in our setting. Despite the high mortality and morbidity of typhoid intestinal perforation in developing world like Tanzania, relatively a little is known about the pattern of this disease and its prognostic factors in our set up. The purpose of this study was to describe our experiences on the surgical management of typhoid intestinal perforation outlining the clinical profile and treatment outcome of this disease and to determine the prognostic factors for morbidity and mortality in our local setting. It is hoped that identification of these factors will help in policy decision making, prioritizing management and improving the quality of care in typhoid intestinal perforation.

## Methods

This was a combined retrospective and prospective study of patients who were operated for typhoid intestinal perforation at Bugando Medical Centre between October 2006 and September 2011. Bugando Medical Centre (BMC) is a consultant, tertiary care and teaching hospital for the Catholic University of Health and Allied Sciences -Bugando (CUHAS-Bugando) with a bed capacity of 1000.

All patients who were operated for typhoid intestinal perforation during the study period were included in the study. Patients with incomplete data and those who failed to consent for HIV infection were excluded from the study. The details of patients who presented from October 2006 to September 2008 were retrieved retrospectively from patient registers kept in the Medical record departments, the surgical wards, and operating theatre. Patients who presented to the A & E department between October 2008 and September 2011 were prospectively enrolled in the study after signing an informed written consent for the study. The diagnosis of typhoid perforation was established by clinical features of typhoid fever and peritonitis which were supported by positive Widal test, detection of free air under the diaphragm on chest and abdominal radiographs and free intra peritoneal fluid on ultrasound abdomen and confirmed by intraoperative findings of oval perforation on the antimesenteric border of the intestine and an acutely inflamed and edematous intestine. Peritonitis was recorded as general when the whole abdomen was involved; it was recorded as local when peritonitis was limited to the lower abdomen. The patients who developed clinical features of peritonitis after typhoid fever and presented within 24 hours were labeled as *early *while those presented after 24 hours were marked as *late *cases. Inadequate prehospital therapy was defined as not being given a minimum of 3 days of effective antibiotic treatment for *S. Typhi *at the correct dose prior to admission. The time of typhoid intestinal perforation was subjectively determined as the time the patient felt an excruciating sharp pain with worsening of symptoms. In small children it was taken as the time the mother noticed abdominal distention, constipation and vomiting.

Preoperatively, all the patients had intravenous fluids to correct fluid and electrolyte deficits; nasogastric suction; urethral catheterization and broad-spectrum antibiotic coverage. Relevant preoperative investigations included packed cell volume, serum electrolytes, urea and creatinine, HIV testing (using Tanzania HIV Rapid Test Algorithm) and CD 4+ count (using FACS or FACSCALIBUR from BD Biosciences USA), Widal's test; chest and abdominal radiographs to detect air under the diaphragm. Abdominal ultrasound was also performed in some patients suspected to have abdominal collections. They had pre-operative anaesthetic assessment using the American Society of Anesthetists (ASA) classification [24] as shown in Table 1. To minimize variability in our study, the assignation of ASA class was performed by one consultant anesthetist adhering strictly to criteria above. After resuscitation all patients under general anaesthesia were subjected to exploratory laparotomy. Adequate hydration was indicated by an hourly urine output of 30 ml/hour. An initial systolic blood pressure (SBP) on each patient was also recorded on admission. Preoperative shock was defined as a preoperative systolic blood pressure of less than 90 mmHg.

**Table 1:** American Society of Anesthetists (ASA) classification

ASA class	Description
I	Healthy individual with no systemic disease

II	Mild systemic disease not limiting activity

III	Severe systemic disease that limits activity but is not incapacitating

IV	Incapacitating systemic disease which is constantly life threatening

V	Moribund, not expected to survive 24 hours with or without operation

*Note*: E is added to the class when the case is an emergency e.g. IIE refers to ASA class scheduled for emergency surgery

Laparotomy was performed by a midline incision; all dirty yellow purulent material was aspirated from peritoneal cavity. General survey of peritoneal cavity was made. In patients with single perforation, the edge of the intestinal perforation was excised, and double-layer closure was done with chromic catgut or coated vicryl 2/0 and silk 2/0. Patients with multiple perforations had bowel resection and anastomosis. Ileostomy and damage control surgery was done in patients with ASA class VE. Copious peritoneal lavage was done with warm isotonic saline, 2 drains were placed, one in the pelvis, the other in the right paracolic gutter, and mass closure of the abdomen was done using nylon-1. The skin was closed with interrupted stitches of nylon-2/0. Post-operatively patients were kept nil orally till return of bowl sounds and at that time nasogastric tubes were removed. IV antibiotics were used for one week. Drains were removed on 6th post operative day. The postoperative outcome was monitored; patients in ASA classes IV and V were admitted into intensive care unit after surgery. Data on each patient were entered into a pro forma prepared for the study. The study variables included socio-demographic data (i.e. age and sex, level of education, occupation and area of residence), clinical presentation, HIV status, radiological findings, perforation-surgery interval, ASA classification, operative findings (such as type of peritonitis, degree of contamination and number of perforations), antibiotics used and surgical procedure performed. The variables studied in the post-operative period were postoperative complications, hospital stay and mortality.

### Statistical analysis

The statistical analysis was performed using statistical package for social sciences (SPSS) version 15.0 for Windows (SPSS, Chicago IL, U.S.A).The mean ± standard deviation (SD), median and ranges were calculated for continuous variables whereas proportions and frequency tables were used to summarize categorical variables. Continuous variables were categorized. Chi-square (χ^2^) test were used to test for the significance of association between the independent (predictor) and dependent (outcome) variables in the categorical variables. The level of significance was considered as *P *< 0.05. Multivariate logistic regression analysis was used to determine predictor variables that predict the outcome.

### Ethical consideration

Ethical approval to conduct the study was obtained from the CUHAS-Bugando/BMC joint institutional ethic review committee before the commencement of the study. Patients recruited prospectively were required to sign a written informed consent for the study and for HIV testing.

## Results

Out of 1213 patients who presented to our centre with typhoid fever during the study period, 123 patients underwent emergency laparotomy for typhoid intestinal perforations. Of these, 19 patients were excluded from the study due to failure to meet the inclusion criteria and incomplete data. Thus, 104 patients were studied giving an average of 10 cases annually and represented 8.5% of cases. Of these, 21 (20.2%) patients were studied retrospectively and the remaining 83(79.8%) patients were studied prospectively.

### Socio-demographic characteristics

**Figure 1:**
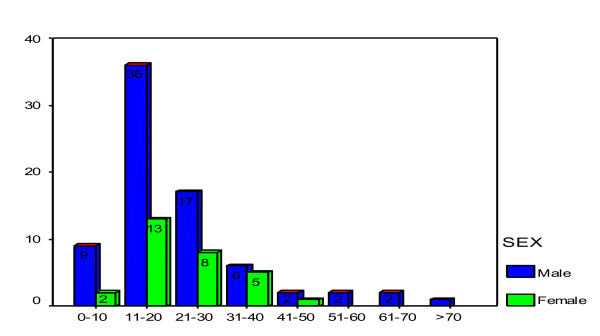
**Age group distribution by sex**.

Seventy- five (72.1%) patients were males and females were 29 (27.9%) with the male to female ratio of 2.6:1. Their ages ranged from 8 to 76 years with a median age of 18.5 years. The peak age incidence was in the 11-20 years age group accounting for 47.1% of cases (Table 2). Figure 1 shows distribution of age group by sex. Most of patients, 86 (82.7%) had either primary or no formal education and more than eighty percent of them were unemployed. The majority of patients, 78 (75.0%) came from the rural areas located a considerable distance from Mwanza City and more than three quarter of them had no identifiable health insurance.

**Table 2:** Distribution of age group by sex

Age group (in years)	Males (N/%)	Females (N/%)	Total (N/%)
0-10	9 (8.7)	2 (1.9)	11 (10.6)

11-20	36 (34.6)	13 (12.5)	49 (47.1)

21-30	17 (16.3)	8 (7.7)	26 (24.0)

31-40	6 (5.8)	5 (4.8)	11 (10.6)

41-50	2 (1.9)	1 (1.0)	3 (2.9)

51-60	2 (1.9)	-	2 (1.9)

61-70	1 (1.0)	-	1 (1.0)

> 70	1 (1.0)	-	1 (1.0)

Total	75 (72.1)	29 (27.9)	104 (100)

### Clinical presentation of patients with typhoid intestinal perforations

Fever and abdominal pain were common to all the patients (Table 3). The duration of illness (fever-perforation interval) was within 14 days in 84 (80.8%) patients and more than 14 days in 20(19.2%) patients. Most patients, 87 (83.7%) had perforation occurred prior to hospital admission, whereas in the remaining 17 (16.3%) patients perforation occurred during the course of hospitalization. Perforation- admission interval was within 24 hours (early presentation) in 16 (15.4%) patients and more than 24 hours (late presentation) in 88 (84.6%) patients. Adequate antibiotic treatment prior to admission was recorded in 26 (25.0%) patients whereas inadequate antibiotic treatment was recorded in 72 (69.2%) patients. Antibiotic treatment prior to admission was not documented in 6 (5.8%) patients. Inadequate treatment prior to admission was significant predictor of intestinal perforation (Odds ratio = 2.3, 95% Confidence interval = 1.4-4.6, *P *= 0.002).

**Table 3:** Clinical features of patients with typhoid

Clinical features	Frequency	Percentage
Fever	104	100

Abdominal pain	104	100

Vomiting	94	90.4

Diarrhea	88	84.6

Constipation	80	76.9

Abdominal distension	76	73.1

Dehydration	72	69.2

Shock	63	60.6

Feculent gastric aspirates	12	11.5

Jaundice	7	6.7

### Investigations

Ninety-nine (95.2%) of the patients had plain abdominal x-ray films available for review and demonstrated free gas under the diaphragm (pneumoperitonium) in 74 (74.7%) of them. Ultrasound done in 56 (53.8%) patients detected free peritoneal collections in 48 (85.7%) patients. Widal's test was positive (i.e. titre ≥ 1 in 160 dilutions) in 98(94.2%) patients. HIV status was known in 88 (84.6%) patients. Of these, 9 (10.2%) were HIV positive. Of the HIV positive patients, four (44.4%) patients were known cases on ant-retroviral therapy (ARV) and the remaining 5 (55.6%) patients were newly diagnosed patients. HIV status was not known in 16 (15.4%) patients. CD 4+ count among HIV positive patients was available in only 7 patients and ranged from 43 cells/μl to 720 cells/μl with the mean of 224 cells/μl and standard deviation of 78 cells/μl. The median and the mode were 261 cells/μl and 172 cells/μl respectively. A total of three HIV positive patients (42.9%) had CD4+ count below 200 cells/μl and the remaining 4 patients (57.1%) had CD4+ count of ≥200 cells/μl. Serum electrolytes revealed hypokalaemia and hyponatraemia in 34 and 21 patients respectively. Histopathological examination of excised specimens from the edges of perforations was typical of chronic inflammation (infiltration by monocytes, lymphocytes, plasma cells) in the 97 (93.3%) patients. Blood and stool cultures were not done in any of the patients

### Anesthetic assessment

All patients were assessed pre-operatively using the American Society of Anaesthetists (ASA) pre-operative grading as shown in Table 4.

**Table 4:** Distribution of patients according to ASA classification

ASA classification	Number of patients	Percentage
I	8	7.7

II	20	19.2

III	40	38.5

IV	31	29.8

V	5	4.8

Total	104	100

### Operative findings

All patients in this study underwent laparotomy. The perforation-surgery interval was within 24 hours in 14 (13.5%) patients and more than 24 hours in 90(86.5%) patients. The interval between presentations at the Accident and Emergency department and surgery (waiting time) ranged from 1-10 hours with a median of 6 hours. On operation the abdominal cavity was heavily contaminated (generalized peritonitis) in 96 (92.3%) patients while in 8 (7.7%) patients the peritoneal cavity was having minimal contamination (localized peritonitis). Eighty-eight (84.6%) had single perforation and the remaining 16 (15.4%) patients had multiple perforations. The median age of the patients with single perforation (45 years) was significantly higher than that of those with multiple perforations (14 years) (*P *= 0.018). A total of 109 perforations were identified and ileum was the most common part of the bowel affected and occurred in 86.2% of cases (Table 5). The median size of the perforations was 7.8 mm (2-28 mm). The median distance from ileocecal junction was 36 cm (range 8-98 cm). The amount of pus/faecal matter drained from the peritoneal cavity reflected the extent of contamination. The drainage was between 200 and 3000 mls with a mean of 628 mls. It was less than 1000 ml in15 (14.4%) patients and more than 1000 mls in 89 (85.6%) patients.

**Table 5:** Distribution of patients according to anatomical site of perforations (N = 109)

Anatomical site	Frequency	Percentage
Jejunum	11	10.1

Ileum	94	86.2

Caecum	2	1.8

Appendix	1	0.9

Ascending colon	1	0.9

Total	109	100

### Surgical procedures

Perforations were surgically treated depending upon the number of perforations, general health status of patient and degree of faecal contamination. Simple closure of the perforations was the most commonly done procedure accounting for 78.8% of cases and this was generally done in two layers after excision the edges (Table 6). Eight (7.7%) patients had re-operation between 3 rd and 14th day post-operatively as follows: 4 (3.8%) patients for intra-abdominal abscess and 2 (1.9%) patients for burst abdomen and enterocutaneous fistula each respectively. Four (3.8%) patients were re-operated during the follow up period as follows: 3 (2.9%) patients underwent Mayo's repair for incisional hernia and 1 (1.9%) patient had laparotomy due to adhesive intestinal obstruction.

**Table 6:** Type of surgical procedures performed (N = 104)

Surgical procedure performed	Frequency	Percentage
Simple double layered closure	82	78.8

Bowel resection with anastomosis	10	9.6

Right hemicolectomy + ileo-transverse anastomosis	8	7.7

Exteriorization of perforation with ileostomy	2	1.9

Appendicectomy	2	1.9

### Clinical outcome

#### Post-operative complications

Forty-one (39.4%) patients had 62 post-complications as shown in Table 7. Surgical site infection was the most common post-operative complication accounting for 55.5% of cases.

**Table 7:** Post-operative complications (N = 62)

Post-operative complications	Response	Frequency	Percentage
***Early postoperative complications***	Surgical site infection	35	55.5

	Chest infections	16	25.8

	Septic shock	5	8.1

	Intra-abdominal abscess	4	6.5

	Enterocutaneous fistula	4	6.5

	Wound dehiscence/burst abdomen	2	3.2

	Post-operative paralytic ileus	2	3.2

	Renal failure	1	1.6

			

***Late postoperative complications***	Adhesive intestinal obstruction	4	6.5

	Incisional hernia	3	4.8

	Hypertrophic/Keloids	2	3.2

#### Length of hospital stay

The overall length of hospital stay (LOS) ranged from 7 to 64 days with a median of 28 days. The median LOS for non-survivors was 6 days (range 1-10 days). Patients who had post-complications stayed longer in the hospital and this was statistically significant (*P *= 0.012).

#### Mortality

In this study, twenty-four patients died giving a mortality rate of 23.1%. Table 8 shows predictors of mortality according to univariate and multivariate logistic regression analysis.

**Table 8:** Predictors of mortality according to univariate and multivariate logistic regression analysis

Independent (Predictor) Variable	Survivors (N/%)	Non-survivors (N/%)	Univariate analysis	Multivariate analysis				
			**O.R**.	**95% C.I**.	***P*-value**	**O.R**.	**95% C.I**.	***P*-value**

**Age**								

≤ 40	77(79.4)	22(22.6)						

> 40	5 (71.4)	2(28.6)	1.23	0.24-2.98	0.984	2.32	0.43-2.45	NS

**Sex**								

Male	58 (77.3)	17(22.7)						

Female	22 (75.9)	7 (24.1)	2.21	0.95-2.76	0.051	1.32	0.22-2.32	NS

**Duration of illness**								

Within 14 days	64 (76.2)	20 (23.8)						

After 14 days	16 (80.0)	4 (20.0)	1.11	0.57-1.98	0.454	1.67	0.78-2.11	NS

**Perforation-admission interval**								

Within 24 hours	15 (93.7)	1 (6.3)						

After 24 hours	65 (73.7)	23 (26.1)	2.43	1.34-3.54	0.024	1.67	1.12-3.43	**0.003**

**Timing of operation**								

Within 24 hours	12(85.7)	2(14.3)						

After 24 hours	68 (75.6)	22(24.4)	0.21	0.11-0.98	0.011	1.23	1.12-3.65	**0.034**

**HIV status**								

Positive	3 (33.3)	6(66.7)						

Negative	63 (79.7)	16 (20.3)						

Not known	14 (87.5)	2 (12.5)	3.54	2.46-4.98	0.031	0.23	0.11-0.98	**0.022**

**CD4+ count (cells/μl)**								

≤ 200	1(33.3)	2 (66.7)						

**> **200	3(75.0)	1(25.0)	5.34	3.45-6.98	0.004	4.54	3.23-6.87	**0.000**

**Prehospital antibiotic therapy**								

Adequate	23 (88.5)	3 (11.5)						

Inadequate	52 (72.2)	20 (27.8)						

Not documented	7 (87.5)	1 (12.5)	2.87	2.11-4.50	0.021	3.11	1.45-7.86	**0.006**

**ASA classes**								

I-II (Low risk group)	26 (92.9)	2 (7.1)						

III-V (High risk group)	54 (71.1)	22 (28.9)	0.32	0.11-0.98	0.033	3.2	2.34-6.81	**0.012**

**SBP on admission**								

≤ 90 mmHg	22 (61.1)	14 (38.9)						

> 90 mmHg	58(85.3)	10 (14.7)	3.45	1.56-4.91	0.011	1.98	1.72-4.98	**0.000**

**Type of peritonitis**								

Generalized	74(77.1)	22 (22.9)						

Localized	6(75.0)	2(25.0)	1.95	0.98-2.75	0.967	0.32	0.11-1.63	NS

**Amount of peritoneal fluid/pus**								

≤ 1000 mls	13 (86.7)	2 (13.3)						

> 1000 ml	67(75.3)	22 (24.7)	1.52	1.18-2.22	0.023	1.22	1.09-1.76	**0.011**

**Number of perforations**								

Single	71 (80.7)	17 (19.3)						

Multiple	9 (56.2)	7(43.8)	1.54	1.11-4.87	0.012	2.89	2.33-5.98	**0.007**

**Postoperative complications**								

Present	25 (61.0)	16 (39.0)						

Absent	55 (87.3)	8 (12.7)	2.98	2.33-4.91	0.004	5.22	3.43-6.94	**0.000**

Keys: N = Number of patients, C.I. = Confidence interval, O.R. = Odds ratio, SBP = Systolic blood pressure

### Follow up of patients

Of the survivors, seventy-six (95.0%) patients were discharged well and the remaining 4 (5.0%) were discharged against medical advice. No patients were discharged with permanent disabilities. Out of 80 survivors, twenty-eight (35.0%) patients were available for follow up at three month after discharge and the remaining 52 (65.0%) patients were lost to follow up.

## Discussion

Intestinal perforation is the most serious complication of typhoid fever in the developing world that presents a challenge to surgeons in that perforation leads to high morbidity and mortality, but development of perforation is also unpredictable [14,15,22-27]. The incidence of the disease varies considerably in different parts of the world [28]. The incidence of typhoid intestinal perforation had previously been reported as an indication of endemicity of typhoid fever in any locality [27,29-34].

In most parts of the world, perforation rate ranges from 0.6% to 4.9% of enteric fever cases [8,35], but in West Africa, higher rates of 10%-33% have been reported [28,29,31,36]. In this review, the rate of typhoid intestinal perforation represented 8.5% of cases which is significantly lower than that reported in Western Africa [29,31,36]. High rate of intestinal perforation in this region may be due to a more virulent strain of *Salmonella typhi *among West Africans, coupled with increased hypersensitivity reaction in the Peyer's patches in this sub-region, where the perforation rate is higher than other endemic areas. These differences in the incidence of the disease reflect differences in the rate of risk factors for typhoid intestinal perforation from one country to another. The figures for the rate of typhoid intestinal perforation in our study may actually be an underestimate and the magnitude of the problem may not be apparent because of high number of patients excluded from this study.

In the present study, the highest incidence of typhoid intestinal perforation occurred in the first and second decades of life which is in keeping with other studies done elsewhere [6,15,28]. The increasing occurrence of typhoid intestinal perforation in this age group in our setting can be explained by the fact that youths are generally more adventurous and mobile and are more likely to eat unhygienic food outside the home. There is also high risk of fecal contamination as they visit the toilets at school or public toilets. High incidence of the disease in this age group has a negative impact on the country's economy because this group represents the economically productive age group and portrays an economic lost both to the family and the nation. The fact that the economically productive age-group is mostly affected demands an urgent public policy response on preventive measures such as safe drinking water and appropriate sewage disposal, and typhoid vaccination.

In agreement with other studies [15,26,27,35,36], typhoid intestinal perforation in the present study was more common in males than in females. The exact reason for this male preponderance is not known although it is possible that men have an increased risk of exposure to typhoid fever resulting from spending longer time and consuming more food outdoors that may lead to more frequent contact with the causative bacteria.

Intestinal perforation resulting from typhoid fever has been reported to be more prevalent in people with low socio-economic status [15]. This observation is reflected in our study where most of patients had either primary or no formal education and more than eighty percent of them were unemployed. The majority of patients in the present study came from the rural areas located a considerable distance from Mwanza City and more than three quarter of them had no identifiable health insurance. Similar observation was reported by others [15,37]. This observation has an implication on accessibility to health care facilities and awareness of the disease.

The clinical presentation of typhoid intestinal perforation in our patients is not different from those in other geographical areas [6,15,26,27,38] with fever and abdominal pain being common to all the patients. In our study, perforation occurred early in the course of the disease and this has been recognized by others [28,29,31,36]. Patients who perforate during the first two weeks of the illness appear to have a better prognosis [36]. It has been observed that compromised nutritional status could possibly play a role in the poor prognosis of the patient who has been ill for more than 2 weeks and then develops a perforation [39], but this observation is yet to be proved. Typhoid intestinal perforation generally occurs in 2nd to 3 rd week of illness, this is because of mechanism of perforation in Peyer's patches of terminal ileum [12] but in developing countries cases are reported early within first week of illness [30], reason behind this observation is not clear but it is speculated to be due to low immune power, change in virulence of bacteria, hypersensitivity to Peyer's patches and ileal contents of bacteria. This observation is reflected in our study where more than fifty percent of patients developed perforation within 1-2 weeks of the illness. The mechanism of intestinal perforation in typhoid fever is hyperplasia and necrosis of Peyer's patches of the terminal ileum. The lymphoid aggregates of Peyer's patches extend from the lamina propria to the sub-mucosa, so that in the presence of hyperplasia the distance from the luminal epithelium to the serosa is bridged by lymphoid tissue. During the course of typhoid fever, *S. Typhi *is found within mononuclear phagocytes of Peyer's patches, and in cases with intestinal perforation, both this tissue and surrounding tissues show hemorrhagic areas, most often during the third week of the illness [3]. Tissue damage in Peyer's patches occurs, resulting in ulceration, bleeding, necrosis, and, in extreme cases, full-thickness perforation. The process leading to tissue damage is probably multifactorial, involving both bacterial factors and host inflammatory response [3,35].

Most patients with typhoid fever who were admitted to our centre, reported after they had perforated which is in keeping with other studies done elsewhere [15,23,28,40]. As reported by many authors [15,40], majority of patients in the present study presented late in poor general condition. This was found to be the most important factor influencing the outcome of surgical procedure as also emphasized by a number of authors [15,23,29,30,36,40]. In resource-poor countries, difficulties in diagnosis, patient transfer, and sub-therapeutic antibiotic treatment often result in delayed presentation to a hospital [3,15,28].

In agreement with other studies [15,23,28,40], the diagnosis of typhoid intestinal perforation in this study was made from clinical evaluation, laboratory investigation, identification of free air under the diaphragm on abdominal and chest radiographs and operative findings such as typical perforation on antimesenteric border, purulent collection and adhesion of bowel loops with friable pussy flecks. The value of the radiological investigation has been compared with other writers and with current radiological techniques; 80-90% of cases are correctly diagnosed. Findings from our study demonstrated free gas under the diaphragm on abdominal and chest radiographs in more than seventy percent of cases which is consistent with other studies [41,42]. A plain abdominal or chest radiograph with free air under the diaphragm is a fairly frequent but variable finding signifying perforated hollow viscus, but its absence does not exclude the diagnosis. Abdominal ultrasonography has also been found to be superior to plan radiographs in the diagnosis of free intra-peritoneal air as confirmed by the present study [43]. For the accurate diagnosis of typhoid intestinal perforation, blood for culture and sensitivity, urine for culture and sensitivity and stool for culture and sensitivity or bone marrow are required. None of these laboratory investigations was performed in our study mainly because of lack of facilities capable of performing these tests. It is very difficult to isolate *Salmonella typhi *from urine and stool specimens in most developing countries. This is often due to lack of culture media, expertise and sometimes previous exposure to inadequate doses of antibiotics. Another major problems relating to the laboratory is the abuse of the Widal's test. It is therefore recommended to carry out studies of baseline value of typhoid agglutinins in our setting as has been done in some areas to know the diagnostic utility of the Widal's test.

The clinical picture of typhoid intestinal perforation may be complex when typhoid fever occurs with HIV infected patients [44]. We could not find any study in the literature that shows the effect of HIV infection on outcome of patients with typhoid intestinal perforation. This observation provides room for research on this topic. The prevalence of HIV infection among patients with typhoid intestinal perforation in the present study, was 10.2% which is higher than 6.5% [45] in the general population in Tanzania. However, the overall HIV seroprevalence in this study may actually be an underestimate and the magnitude of the problem may not be apparent because many patients were excluded from the study due to failure to meet the inclusion criteria. We could not establish the reason for the high seroprevalence of HIV among these patients although it is possible that these patients have an increased risk of exposure to HIV infection. This calls for a need to research on this observation. HIV infection was found to be associated with poor postoperative outcome. This observation calls for routine HIV screening in patients suspected to have typhoid intestinal perforation.

Surgical intervention is considered to be the standard treatment of choice for patients with typhoid intestinal perforation [16,46]. In keeping with other studies [4,6,12-15,25-28,33], all patients in the present study underwent surgical treatment. One of the many factors affecting the surgical outcome in patients with typhoid intestinal perforation is time interval between duration of illness and surgical intervention (perforation-surgery interval) [46,47]. Early surgery can minimize the complications while delayed surgery leads to severe peritonitis and septic shock. In the present study, the majority of patients were operated more than 24 hours after the onset of illness. Similar observation was reported by other studies done in developing countries [47]. Delayed definitive surgery in the present study may be attributed to late presentation due to lack of accessibility to health care facilities, lack of awareness of the disease as a result some patients with typhoid perforation may decide to take medications in the pre-hospital period with hope that the symptoms will abate. It is also possible that some clinicians managing the patients initially may not have considered perforation as a possible diagnosis. In resource-poor countries, difficulties in diagnosis, patient transfer, and inadequate antibiotic treatment often result in delayed presentation to a hospital [3,36].

The presence of single intestinal perforations in majority (84.6%) of our patients is consistent with other reports [6,15,29,30]. The median age of the patients with single perforations in the present study was significantly higher than that of those with multiple perforations which is line with other reporters [38,47]. We could not establish the reason for this observation. The number of intestinal perforation in patients with typhoid intestinal perforation has been reported to have an influence on prognosis. In the present study, patients with multiple perforations had significantly high mortality rates compared to those with single perforations. Beniwal *et al *[46] found that the number of perforation had effect on surgical outcome. Adesunkanmi *et al *[36] reported high incidence of residual abscess in patients with single perforation, Kaybal *et al *[48] reported better outcome in single perforation and worse prognosis in multiple perforations while Rehman [31] does not favor correlation of perforation to morbidity and mortality.

Although typhoid ulcers could occur anywhere from the stomach to the rectum [22], the terminal ileum is usually mostly involved due to the high concentration of Payer's patches. Whereas the ileum was the most common site of typhoid perforation in the present study, colonic involvement was very rare which is consistent with other studies [12,15,22,23,25,26,28,32,37]. It is postulated that colonic involvement is due to direct bacteria invasion while ileal lesions are due to enterotoxin produced from parasitizes macrophages that caused hyperplasia, necrosis and ulceration [49].

Early surgical interference is the optimal treatment option for perforation. However, the type of surgery to be applied is controversial. Many surgical techniques have been used, ranging from simple peritoneal drainage under local anaesthesia in moribund patients [15], excision of the edge of the ileal perforation, and simple transverse closure in two layers; as done for majority of our patients, segmental intestinal resection and primary anastomosis especially in multiple perforations or right hemicolectomy where the caecum is involved. Whereas, better results are reported with simple closure, in many series [15,25,26,38,39,41], others favour segmental ileal resection and anastomosis [50]. Those that favour simple closure argue, that in such very ill patients any prolonged procedure may jeopardize the outcome and that the ileum affected by typhoid fever, take sutures well without cutting through. Our practice in managing these patients is a simple closure in solitary perforations and segmental intestinal resection and primary anastomosis in multiple perforations, right hemicolectomy where the caecum is involved and ileostomy for severe peritoneal contamination. The role of ileostomy as a first line operation for typhoid perforation continues to be debated. It has been recommended for patients with severe peritoneal contamination; enhancing intestinal decompression with improved healing, early resolution of ileus and early start to enteral feeding [23,27]. The major drawback of ileostomy is the need for a second operation to restore intestinal continuity, the specialized care before closure and the attendant cost which reduces its popularity [27]. The challenge is even more conspicuous in a developing country like Tanzania where resources for caring of patients with ileostomy are limited. The management of stoma remains difficult in developing countries because of the shortage of suitable equipment in this respect and peristomal ulceration remains a major problem. Indeed, peristomal ulceration provokes skin pain, inducing the patient to self-limitation of food intake leading to severe malnutrition.

The use of antibiotics has been extensively discussed in the past. Chloramphenical with metranidazole used to be the antibiotic of choice [6,15] and is still used in some centers [23]. With increasing resistance of the organisms to Chloramphenical, Cephalosporins (e.g. Ceftriaxone) and Quinolones (e.g. ciprofloxacin) came into being with metranidazole added for the anaerobes and gentamicin for the gram-negative pathogens. This is the regimen commonly used in our centre. However, a recent study done in our centre has shown resistance of the organisms to this combination and highly sensitive to Imipenem and meropenem [51]. But unfortunately these drugs may not be readily available in many third world countries including Tanzania.

The overall complications rate in this series was 39.4% which is comparable to what was reported by others [13,23]. High complications rate was reported by Kouame *et al *[27]. This difference in complication rates can be explained by differences in antibiotic coverage, meticulous preoperative care and proper resuscitation of the patients before operation, improved anesthesia and somewhat better hospital environment. In agreement with other studies [6,13,15,28,37], surgical site infection was the most common postoperative complications in the present study. High rate of surgical site infection in the present study may be attributed to contamination of the laparotomy wound during the surgical procedure.

The overall median duration of hospital stay in the present study was 28 days which is higher than that reported by other authors [15,22,23,25,31]. This can be explained by the presence of large number of patients with postoperative complications in our study.

In the developing world, mortality rates from typhoid perforation have been reported to range from 9-22%. The mortality rate of 23.1% in the present study is comparable to the rates reported from tropical countries such as 22.0% from Nigeria where chloramphenical is still the drug of first choice [14]. These figures are much higher than the rates reported from other tropical countries such as 6.8% from Nepal [52], and 10.5% from India [46]. A high mortality rate of 39.0% was also reported in Nigeria by Meier *et al *[53]. Exceptionally low mortality rates of 1.5-2% have been reported from some parts of the developed world, where socioeconomic infrastructures are well developed [21]. The reasons for the high mortality are multifactorial. In our experience in this study high mortality rate was attributed to delayed presentation, inadequate antibiotic treatment prior to admission, shock on admission, HIV positivity, low CD4 count (< 200 cells/μl), high ASA classes (III-V), delayed operation, multiple perforations, severe peritoneal contamination and presence of postoperative complications.

Self discharge by patient against medical advice is a recognized problem in our setting and this is rampant, especially amongst surgical patients. Similarly, poor follow up visits after discharge from hospitals remain a cause for concern. In the present study, only 35% of survivors were available for follow up which is in keeping with other studies done in developing countries [15]. These issues are often the results of poverty, long distance from the hospitals and ignorance.

The potential limitation of this study is the fact that information about some patients obtained retrospectively was incomplete and this might have introduced some bias in our findings. Also, data obtained retrospectively and failure to detect HIV infection during window period may have underestimated the prevalence of HIV infection in our study. However, despite these limitations, the study has highlighted our experiences with typhoid intestinal perforation and their outcome of surgical management in our limited-resource environment and has provided local data that can guide health care providers in the treatment of patients. The challenges identified in the management of these patients in our setting need to be addressed, in order to deliver optimal care for these patients and improve their treatment outcome.

## Conclusion

Typhoid intestinal perforation is still endemic in our setting and carries high morbidity and mortality. Delayed presentation, inadequate antibiotic treatment prior to admission, shock on admission, HIV positivity, low CD4 count (< 200 cells/μl), high ASA classes (III-V), delayed operation, multiple perforations, severe peritoneal contamination and presence of postoperative complications were the main predictors of mortality in this study. Early and appropriate surgical intervention, effective perioperative resuscitation, postoperative intensive care procedures, safe anesthesia, and delivery of wide-spectrum antibiotics with low resistance are highly recommended in the management of typhoid intestinal perforation in this region. Emphasis should be on preventive measures such as safe drinking water and appropriate sewage disposal, and typhoid vaccination.

## Competing interests

The authors declare that they have no competing interests.

## Authors' contributions

PLC contributed in study design, literature search, data analysis, manuscript writing, editing and submission of the manuscript. JBM, MK, HJ, SEM, MM and GG participated in study design, data analysis, manuscript writing & editing. MDM participated in data analysis, literature search, manuscript writing & editing. JMG supervised the study and contributed in data analysis, manuscript writing & editing. All the authors read and approved the final manuscript.
